# Reactive Oxygen Species, Metabolic Plasticity, and Drug Resistance in Cancer

**DOI:** 10.3390/ijms21103412

**Published:** 2020-05-12

**Authors:** Vikas Bhardwaj, Jun He

**Affiliations:** 1 College of Pharmacy, Thomas Jefferson University, Philadelphia, PA 19107, USA; vikas.bhardwaj@jefferson.edu; 2 Department of Pathology, Anatomy & Cell Biology, Sidney Kimmel Medical College, Thomas Jefferson University, Philadelphia, PA 19107, USA

**Keywords:** reactive oxygen species, metabolic adaptation, drug resistance, cancer

## Abstract

The metabolic abnormality observed in tumors is characterized by the dependence of cancer cells on glycolysis for their energy requirements. Cancer cells also exhibit a high level of reactive oxygen species (ROS), largely due to the alteration of cellular bioenergetics. A highly coordinated interplay between tumor energetics and ROS generates a powerful phenotype that provides the tumor cells with proliferative, antiapoptotic, and overall aggressive characteristics. In this review article, we summarize the literature on how ROS impacts energy metabolism by regulating key metabolic enzymes and how metabolic pathways e.g., glycolysis, PPP, and the TCA cycle reciprocally affect the generation and maintenance of ROS homeostasis. Lastly, we discuss how metabolic adaptation in cancer influences the tumor’s response to chemotherapeutic drugs. Though attempts of targeting tumor energetics have shown promising preclinical outcomes, the clinical benefits are yet to be fully achieved. A better understanding of the interaction between metabolic abnormalities and involvement of ROS under the chemo-induced stress will help develop new strategies and personalized approaches to improve the therapeutic efficiency in cancer patients.

## 1. Introduction

Otto Warburg first observed alterations in cancer metabolism, wherein cancer cells produce most of their energy through glycolysis rather than mitochondrial oxidative phosphorylation in the presence of oxygen, in the 1920s [1]. Warburg observed that tumor cells convert majority of the glucose into lactate and not into CO_2_ as observed in non-tumorous mammalian cells. The observation has since been witnessed by various other researchers and has been thoroughly reviewed [2,3]. Although glycolysis is an inefficient mechanism of energy production (glycolysis generates two ATP molecules, whereas the tricarboxylic acid (TCA) cycle produces 34 ATPs per glucose molecule), it provides cancer cells with ATP at a considerably faster rate than through mitochondria [4,5]. For quicker energy production, a cancer cell can enhance their uptake of glucose, a property commonly employed to visualize tumors using fluorodeoxyglucose, a radio-labeled glucose during positron emission tomography [6]. The upregulation of glycolysis also supports the proliferating cells by providing metabolites, such as serine, glycine, and alanine, for their anabolic processes [7,8,9]. Alternatively, the glycolytic metabolites can also be shunted into the pentose phosphate pathway (PPP) and provide cancer cells with ribose nucleotides and redox potential. Since the microenvironment provides tumor cells with ample nutrients (e.g., glucose, glutamine), ATP produced via glycolysis is sufficient to fulfill the tumor’s requirements. The importance of glycolysis can be appreciated by studies demonstrating that its inhibition suppresses ATP production and leads to cancer cell apoptosis [10,11,12].

The Warburg effect shifts cancer cells from oxidative to reductive metabolism. The reductive metabolism is imperative for the biosynthesis of amino acids and metabolites to sustain cancer survival and growth. The shift in metabolism reduces the dependence on mitochondrial citrate and ATP as these are detrimental to the survival of cancer cells [13,14,15,16]. Genetic and epigenetic regulation of TCA cycle enzymes observed in cancer further support the metabolic shift away from mitochondrial respiration. The altered metabolism is widely considered as one of the hallmarks of cancer, and emerging evidence supports its key role in tumor development through its interaction with other drivers of tumor, namely oncogenes, tumor suppressors, and cellular redox balance [17].

Reactive oxygen species (ROS) are oxygen-containing and chemically reactive species formed by incomplete one-electron reduction of oxygen, which includes hydrogen peroxide (H_2_O_2_), superoxide anion (O_2_^−^), and hydroxyl radical (OH^−^) [18]. ROS are naturally produced in cells through aerobic metabolism. Mitochondria respiratory chain, NADPH oxidase, and peroxisomes are the major endogenous sources of ROS. Under physiological conditions, normal cells maintain redox homeostasis with a low level of basal ROS by controlling the balance between ROS generation (pro-oxidants) and elimination (antioxidant capacity). A moderate increase in ROS favors cell proliferation and survival. However, when the amount of ROS reaches a certain level, it may overwhelm the antioxidant capacity of the cell and trigger cell death by oxidizing cellular macromolecules such as proteins, nuclear acids, and lipids. Growing evidence suggests that cancer cells exhibit increased intrinsic ROS stress due to metabolic abnormalities and oncogenic signaling. In order to maintain the redox dynamics of high ROS, cancer cells trigger an adaptation response by upregulating their antioxidant capacity.

**Figure 1:**
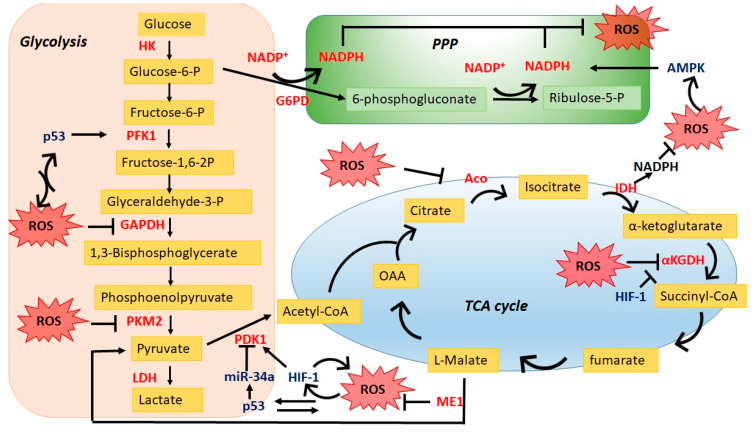
Interplay between reactive oxygen species (ROS) and the central carbon metabolism. ROS impact cellular metabolism through regulating key enzymes in glycolysis pathway/TCA cycle as well as redox signaling pathways; reciprocally, energy metabolic pathways especially PPP balance ROS homeostasis in cancer cells. PPP, pentose phosphate pathway; HK, hexokinase; PFK1, phosphofructokinase 1; PDK1, pyruvate dehydrogenase kinase; G6PD, glucose 6-phosphate dehydrogenase; Aco, aconitase; αKGDH, Alpha ketoglutarate dehydrogenase; IDH, isocitrate dehydrogenase; ME1, malic enzyme 1.

The generation and maintenance of ROS homeostasis in cells largely rely on cellular metabolism while ROS also impacts energy metabolism by regulating key metabolic enzymes and critical oncogenic signaling pathways. In this review, we highlight studies that underline the interplay between the cellular redox balance and tumor metabolism, and explore how these mechanisms support tumor survival under drug-induced stresses (Figure 1).

## 2. ROS Impacts Cancer Metabolic Reprogramming

### 2.1. Direct Regulation through Key Metabolic Enzymes

Although the Warburg hypothesis postulated elevated glycolysis in proliferating cells, the overall metabolic regulation in cancers is rather complex. In addition to glycolysis, cancer cells demonstrate elevated flux into the pentose phosphate pathway, enhanced glutamine consumption, enhanced rate of lipid biosynthesis, and utilization of protein as a fuel source [19,20,21]. These metabolic deregulations however do not occur in silo. It interacts with numerous signaling molecules to promote tumor phenotype. One such deregulation commonly observed in cancer cells is the elevated ROS level [22]. ROS plays a crucial role in maintaining and promoting the tumor phenotype via regulating oncogenic signaling and cellular metabolism [23,24,25]. The ROS levels however are to be kept within a certain range since high ROS levels can be detrimental to cancer survival [26]. Below, we have discussed how the cellular ROS and redox mechanism regulate tumor metabolism with a focus on central carbon metabolism, namely glycolysis, PPP, and the TCA cycle.

#### 2.1.1. Glycolysis

Recent evidence suggests that upregulated NADPH oxidase (NOX) shifts the tumor’s metabolism towards glycolysis in cells with mitochondrial dysfunction [27]. The uncovering of the novel role of NOX is notable since NOX catalyzes the conversion of molecular oxygen to the superoxide ion (O_2_ → O^−^), and its upregulation is commonly observed in tumors [28].

Pyruvate kinase M2 (PKM2) is a rate-limiting glycolytic enzyme that converts phosphoenolpyruvate (PEP) and ADP to pyruvate and ATP [29]. The enzyme pyruvate kinase (PK) exists in two different isoforms: M1 and M2. Under physiological condition, the M1 isoform (PKM1) predominates, whereas cancer cells primarily express the M2 isoform (PKM2) [30]. PKM2 has significantly less pyruvate kinase activity, and it prevents the flow of glycolytic metabolites into the TCA cycle. The metabolites thus accumulated are utilized to meet the biosynthetic needs of the cancer cell [31]. PKM2 also promotes the Warburg effect by activation of the HIF-1α target genes *SLC2A1*, *LDHA,* and *PDK1* that facilitate the shift from oxidative phosphorylation to glycolytic metabolism to meet the nutrient demands of cancer cell proliferation [32]. PMK2 is highly expressed in various cancers, including lung, breast, and prostate, indicating a critical role in cancer progression beyond glycolysis [33]. PKM2 also represents one of the best examples of how ROS can directly regulate cellular metabolism. One study found that an increase in cellular ROS levels by hydrogen peroxide significantly reduced the pyruvate kinase activity of PKM2 through oxidation of Cys^358^. However, this reduced pyruvate kinase activity recovered in the presence of a reducing agent, confirming that PK inhibition in the presence of hydrogen peroxide is ROS-dependent [25]. Further, the inhibition of pyruvate kinase activity promotes CO_2_ production by the PPP and increases the production of reduced glutathione (GSH). The reduced pyruvate kinase activity thus promotes channeling of glycolytic metabolites into the PPP, which in turn increases GSH production to counter elevated ROS [25]. Mutation of Cys^358^ residue prevents ROS-induced inhibition of pyruvate kinase activity, leading to reduced GSH levels and sensitization of the cells to oxidative stress. Similarly, insulin-induced ROS inhibits pyruvate kinase activity in hepatocellular carcinoma [34,35]. Reduced pyruvate kinase activity was observed despite the induction of PKM2 protein levels in cells treated with insulin through suppression of miR-128 and miR-145 [34]. Although unclear, suppression of miR-128 and miR-145 may involve ROS induced DNA hypermethylation [36].

Glyceraldehyde 3-phosphate dehydrogenase (GAPDH) is another glycolytic enzyme readily regulated by the cellular redox system. It catalyzes the conversion of glyceraldehyde 3-phosphate to 1,3-diphosphoglycerate. GAPDH is often regarded as a housekeeping gene and used as reference control. However, its expression is upregulated in a wide variety of tumors and is associated with tumor proliferation, metastasis, and overall aggressive tumor behavior [37,38,39,40]. The elevated GAPDH levels are considered essential to maintaining the glycolytic phenotype present in tumors. Early evidences show that accumulation of ROS is associated with reduced GAPDH activity [41,42]. In addition, treatment with oxidizing low-density lipoprotein reduced the expression of GAPDH in a ROS-dependent manner by increasing its proteosomal-mediated degradation [43]. Mechanistic analysis reveals that oxidizers, such as hydrogen peroxide, nitric oxide, and peroxides, cause oxidization of free cysteine thiols present on GAPDH [44,45,46]. The ROS induced GAPDH inhibition alters the function of GAPDH, leading to redirecting of glycolytic metabolites towards the PPP [37]. Since cancer cells usually express higher ROS levels than non-transformed cells, the alteration of expression and activity of glycolytic enzyme such as PKM2 and GAPDH may represent a necessary adaptation to enhance reducing power of the tumor cells by redirecting the metabolites into PPP for production of NADPH.

#### 2.1.2. TCA Cycle

The tricarboxylic acid cycle plays an essential role in energy production, macromolecule synthesis, and maintenance of cellular redox balance. The TCA cycle through its series of biochemical reactions utilizes oxidized glycolytic product (acetyl CoA) to generate ATP, NADH, and FADH2. The electrons released from NADH and FADH2 enter the electron transport chain (ETC), where the electron is utilized to synthesize ATP in the presence of oxygen. The electron transport chain serves as the primary source of ROS in the cells (discussed below). Accumulating evidence uncovers the critical role of cellular redox status in direct or indirect regulation of TCA cycle activity. Mass spectrometry-based central carbon metabolic analysis reveal that induction of ROS by vitamin C inhibit the levels of various TCA cycle metabolites in breast cancer cells [47]. Few of the enzymes that are regulated by ROS are discussed below.

The enzyme aconitase (Aco) catalyzes the conversion of citrate to isocitrate. It has been demonstrated that the activity of enzyme Aco is often deregulated in cancers either due to mutation or reduced expression [48,49,50]. Aconitase is vulnerable to reactive oxygen and reactive nitrogen species [51,52,53]. The iron-sulfur cluster present in the aconitase enzyme is highly susceptible to oxidation, leading to the iron release and consequently, inactivation of the enzyme. Although the underlying benefit of ROS-induced Aco inactivation is still being uncovered, a recent study demonstrated that overexpression of Aco weakens Warburg-like features in breast cancer cells [54].

Alpha ketoglutarate dehydrogenase *(*αKGDH or 2KG) is a highly regulated TCA cycle enzyme that catalyzes conversion of α-ketoglutarate and coenzyme A to succinyl coA and in the process converts NAD^+^ to NADH. Initial studies with cardiac mitochondria demonstrated that ROS inhibit NADH production and oxidative phosphorylation. Mechanistic analysis revealed that reduced mitochondrial activity observed was associated with reduced activity of αKGDH [55,56]. Although the studies assessing the effect of ROS on αKGDH in cancers are lacking, inhibition of αKGDH promotes utilization of glutamine derived αKG for fatty acid synthesis [57,58]. The citrate synthesized via reductive metabolism of αKG is important for viability and cancer biomass increase [59,60]. The activity of enzyme αKGDH is also inhibited by HIF-1, thus contributing to the reductive carboxylation of αKG [60].

### 2.2. Indirect Regulation through Oncogene or Tumor Suppressor Networks

Accumulating research evidence suggests that driver gene mutations found in cancers contribute to cell metabolic alterations, indicating that signaling pathways may influence the metabolic shift in cancer [61]. It has been revealed that ROS may control tumor cell metabolism by oxidation of oncogenes or tumor suppressors.

#### 2.2.1. AMP-Activated Protein Kinase (AMPK)

AMPK is a key protein to control cellular energy homeostasis, which is generally a negative regulator of the Warburg effect. AMPK is activated by insufficient fuel supply and low oxygen to make nutrients for the anabolic/growth-promoting metabolic pathway [62]. As a stress-response molecule, AMPK acts as a tumor suppressor to prevent the carcinogenesis as a canonical downstream effector of LKB1. However, once tumors develop, AMPK becomes a tumor promoter by protecting against metabolic, oxidative, and genotoxic stresses, and is involved in cancer drug resistance [63]. AMPK is closely linked to redox homeostasis. Reduced nicotinamide adenine dinucleotide phosphate (NADPH) provides reducing power in many enzymatic reactions and also acts as an antioxidant to neutralize ROS [64]. AMPK regulates NADPH homeostasis by inhibition of the acetyl-CoA carboxylases ACC1 and ACC2, thus decreasing NADPH consumption and increasing NADPH generation where the pentose phosphate pathway is impaired [65]. AMPK itself is redox active in that it contains cysteine residues that can be oxidized by ROS. An earlier study showed that exposure to H_2_O_2_ can activate AMPK through the redox-sensitive cysteine residues (Cys-299/Cys-304) in the α1 catalytic subunit [66]. The mitochondrial ROS mediated AMPK activation is sufficient to mediate starvation-induced autophagy [67]. A recent study argued that mitochondria-derived ROS indirectly affects AMPK activity by decreasing the ATP/ADP ratio rather than the direct protein thiol oxidation [68].

#### 2.2.2. Hypoxia-Inducible Factor 1 (HIF-1)

Hypoxia is a characteristic feature of solid tumors due to an imbalance between oxygen (O_2_) supply and consumption, in which HIF-1 is a key regulator in response to low oxygen [69,70]. The activation of HIF-1 by hypoxia modulates erythropoiesis and angiogenesis, as well as glycolytic metabolism through multiple target genes. Many key glycolytic proteins are HIF-1 transcriptional target gene products, including glucose transporter 1 and 3 (GLUT1 and GLUT3), hexokinase (HK), 6-phosphofructo-2-kinase/fructose-2,6-bisphosphatases 3 (PFKEB3), and pyruvate kinase M2 (PKM2). Induction of these genes by HIF1 enhances glycolysis and the PPP pathway [71]. The overall metabolic outcome of HIF1 upregulation in cancer is to promote aerobic glycolysis. In addition to upregulation of glucose uptake, HIF-1 transcriptionally activates expression of pyruvate dehydrogenase kinase (PDK) [72]. The PDK inhibits the activity of pyruvate dehydrogenase, thus limiting the entry of glycolytic metabolites in the TCA cycle [72,73]. In addition, HIF-1 mediated metabolic reprogramming involves a reduction in cellular ROS levels via inhibition of electron transport chain complex 1 activity [74,75]. Studies show that HIF-1 is activated not only by hypoxia but also by growth factors and oncogenes. Our group previously found that ROS scavengers and antioxidant enzymes decreased HIF-1α expression levels in a dose-dependent manner under normoxic conditions in ovarian cancer cells [76]. Further studies revealed that heavy metals- or growth factors-induced HIF-1α activation in normoxia is mediated by cellular ROS production [77,78]. Jung et al. found that adenosine monophosphate-activated protein kinase (AMPK) mediates ROS-induced HIF-1α protein accumulation at the post-translational step by blocking its degradation resulting from the ubiquitination inhibition [29]. They also suggested that H_2_O_2_ might increase the transcriptional activity of HIF-1α through AMPK. Notably, the role of AMPK in hypoxia-induced HIF-1 activation is different than that in ROS-induced HIF-1 activation.

#### 2.2.3. p53

p53 plays a key role in maintaining genome integrity in response to cellular stresses that lead to DNA damage. It is well-established that p53 acts as a negative regulator of glycolysis by inhibiting expression levels of glucose transporters, which limits activities of glycolytic enzymes phosphofructokinase 1 (PFK1) and phosphoglycerate mutase (PGM) [36]. There is a direct interplay between p53 and ROS leading to oxidative stress. On the one hand, p53 modulates cellular ROS levels through p53-inducible genes (PIGs) that encode a number of pro-oxidant enzymes to generate ROS or inhibit antioxidant genes, such as MnSOD, at the transcriptional level [79]. On the other hand, p53 contains cysteine (Cys) residues in its DNA binding domain, which can be oxidized by ROS. The oxidation of cysteines would impair the DNA-binding activity of p53 to specific genes. ROS can also activate protein kinases such as mitogen-activated protein kinase (MAPK), which in turn phosphorylate and thus activate p53 for apoptosis induction [80].

#### 2.2.4. ROS-Responsive miRNAs

Deregulations of microRNA expression have been associated with tumor development, progression, metastasis, and therapeutic responses [81]. Notably, miRNAs are shown to mediate metabolic phenotypes through the regulation of glycolytic enzymes and mitochondria metabolism [82]. Growing evidence suggests a reciprocal connection between ROS signaling and the microRNA pathway, resulting in diverse biological effects in cancer cells [83]. Altered productions of ROS are associated with deregulated expression of miRNAs, suggesting that miRNAs play a role in regulating ROS production and vice versa. ROS control miRNAs expression levels at multiple layers. The proposed mechanisms include miRNA biogenesis, transcription, and epigenetic regulation [83]. For example, activation of miR-34a switches the glycolysis to mitochondria respiration in cancer cells by direct targeted-inhibition of glycolytic enzymes (e.g., hexokinase 1 (HK1), hexokinase 2 (HK2), glucose-6-phosphate isomerase, and pyruvate dehydrogenase kinase 1 (PDK1)) in cancer cells inp53-dependent manner [84]. As p53 is involved in microRNA processing pathways, such as Drosha-mediated pri-microRNA processing, ROS indirectly affect the miR-34a levels by promoting the transcription of the miR-34a gene through p53.

## 3. Glucose Metabolic Adaptation Alters the Redox Balance

As mentioned earlier, the mitochondrial electron chain and NOX are the main source of cellular ROS. In cancer cells, the high ROS levels are countered by enhanced antioxidant capacity of the cells [85]. The critical balance between ROS and antioxidant mechanisms is essential for cellular homeostasis as different ROS levels can initiate varied biological responses ranging from cellular signaling to oxidative damage of cellular proteins and genomic instability [86]. As expected, the ROS do not control varied cellular functions independently. ROS interact with cellular oncogenes and tumor suppressors and have complex interplay with tumor metabolism. As highlighted above, the mitochondrial citrate and ATP production is detrimental to cancer cells. In a similar vein, the mitochondrial ROS production is also significantly reduced to maintain ROS at a non-toxic level [87]. Below we highlight the accumulating evidence that emphasizes the role of metabolic pathways in regulating cellular ROS levels.

### 3.1. Glycolysis

ROS can regulate the expression of glycolytic enzymes, such as PKM2 and GAPDH. Recent studies have also elaborated the essential role of glycolysis in regulating cellular ROS levels. The enzyme lactate dehydrogenase A (LDHA) converts glycolytic pyruvate into lactate and is upregulated in various tumors [88]. siRNA mediated inhibition of enzyme LDHA diverting the pyruvate into TCA [89,90]. The inhibition of TCA flux by LDHA thus prevents the generation of mitochondrial ROS.

### 3.2. Pentose Phosphate Pathway

The most well understood tumor metabolic regulation that alters the cellular redox balance is the upregulation of the PPP. The PPP branches from glycolysis and provides the proliferating cancer cells with nucleotides (non-oxidative PPP) and NADPH (oxidative PPP). Cancer cells display elevated levels and activity of PPP enzymes involved in oxidative PPP, namely glucose 6-phosphate dehydrogenase (G6PD) [91,92,93,94]. The G6PD is the primary source of cellular NADPH and is upregulated in various tumors [95]. The importance of G6PD can be gauged through its essential role in cellular growth, neoplastic transformation, and tumorigenesis [96,97]. G6PD is the rate-limiting enzyme that controls the entry of glycolytic glucose-6-phosphate into the PPP. Since the NADPH plays a crucial role in reducing cellular oxidants, such as hydrogen peroxide (H_2_O_2_) and other ROS species [98], the cellular antioxidant system is directly or indirectly dependent on NADPH for its functioning. The enzyme glutathione reductase reduces glutathione (GSH) in the presence of NADPH [99]. The GSH is an essential antioxidant defense mechanism in the cell that converts H_2_O_2_ into water. Other than glutathione, the antioxidant activity of nitric oxide synthetase is also dependent on NADPH derived from the oxidative PPP, namely the enzyme G6PD [100,101].

### 3.3. Tri-Carboxylic Acid (TCA) Cycle

As outlined above, the TCA cycle is a major source of cellular reducing equivalent that transfers electrons to the electron transport chain (ETC). However, up to 2% of electrons leak out of ETC and interact with mitochondrial oxygen, leading to the formation of superoxide ions, which can ultimately result in the formation of hydrogen peroxide and hydroxyl and peroxynitrite radical [102,103,104,105,106]. Hydroxyl radical and peroxynitrite are strong oxidants that can interact with cellular components leading to signaling, or oxidation of lipids, proteins, and DNA in a concentration-dependent manner [107,108,109].

The reduced oxidative phosphorylation in cancer does mean that the TCA is non-functional [110]. Recent studies have demonstrated that the TCA cycle continues to generate essential macromolecules and energy by glutamine-induced anaplerosis [111,112,113]. The intracellular glutamine is converted into α-ketoglutarate, which is then converted into citrate by the activity of enzyme isocitrate dehydrogenase (IDH). The synthesized citrate serves as a precursor for the synthesis of fatty acids and other macromolecules for tumor anabolic processes [58,114].

The isocitrate dehydrogenase enzyme (IDH) is expressed in three isoforms where IDH1 is found in cytoplasm and IDH2 and IDH3 are mitochondrial bound [115]. Of these three, the IDH3 predominates at the TCA cycle, is NAD^+^ dependent, and catalyzes irreversible oxidative decarboxylation of isocitrate (ICT) in the presence of NAD^+^ to produce 2KG and NADH. IDH1 and IDH2, on the other hand, are NADP-dependent and can catalyze in both reactions (ICT to 2KG and vice versa). The two NADP-bound IDH enzymes are essential for synthesis of NADPH and play a crucial role in cellular defense against oxidative injury [116,117,118]. Antisense mediated inhibition of NADP-dependent IDH (IDH1 and IDH2) significantly enhanced γ-radiation-induced ROS production, lipid peroxidation, and protein oxidation [119]. Analysis of TCGA data demonstrates that IDH1 is upregulated in several malignancies, including anaplastic large cell lymphoma, glioblastoma, and pancreatic ductal adenocarcinomas, and it is associated with poor prognosis among leukemia patients [120,121,122,123]. Knockdown of IDH1 significantly reduces NADPH content, leading to reduction in GSH levels and induction of ROS in glioblastoma cells. Recently, mutant forms of IDH have been observed in a variety of malignancies, including glioblastomas, leukemias, osteosarcomas, and thyroid tumors [124,125,126,127]. The enzymatic activity of mutated IDH1/2 displays conversion of 2KG into an oncometabolite D-2-hydroxyglutarate and, in the process, IDH1/2 consumes NADPH. Overexpression of mutant IDH1 (R132H) in glioblastoma cells reduces NADPH and GSH levels leading to elevated ROS levels [128]. The R132H mutant IDH1 also has a dominant negative affect on IDH1 activity that may contribute to reduced NADPH and elevated ROS levels [129]. Though the production of oncometabolites by R132H mutants enhances HIF1α levels and induces tumor formation, the reason for the R132H mutation being associated with improved glioma patient survival remains elusive [128,129,130,131].

Malic enzymes (ME) catalyze the conversion of malate into pyruvate and, in the process, synthesize reducing equivalents [132]. ME is encoded by three homologous genes, of which ME1 is located in the cytoplasm, whereas ME2 and ME3 are present in mitochondria. The ME1 is an NADP bound enzyme and, along with G6PD, is the major source of cellular NADPH [133,134]. Recent reports have suggested that ME1 behaves as an oncogene and is associated with tumor growth and invasion [135,136]. Investigation of the role of ME1 in gastric cancer reveals that knockdown of ME1 is associated with elevated ROS and decreased NADPH levels in glucose limiting conditions [137].

## 4. Metabolic Deregulations Lead to Drug Resistance

**Figure 2:**
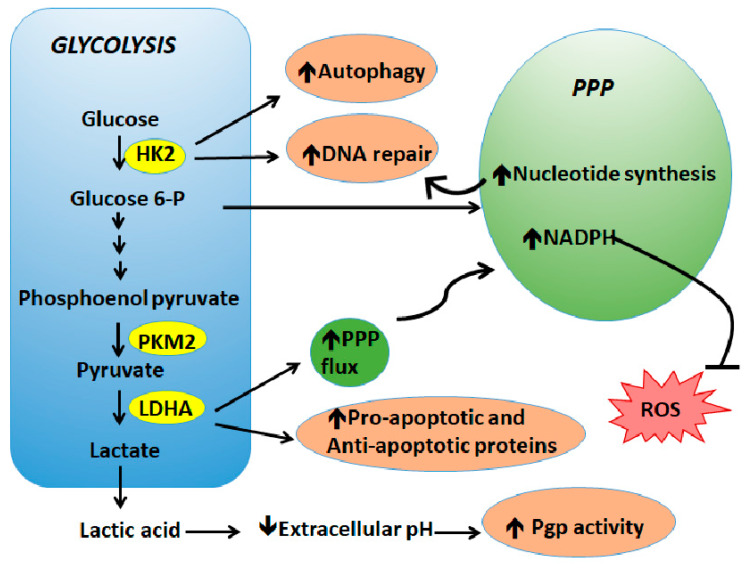
Metabolic deregulations lead to drug resistance. The metabolic shift favors cancer cell proliferation and survival in response to therapy via upregulation of DNA repair, increase of prosurvival signaling and autophagy, activation of drug efflux pumps, and neutralization of ROS.

Overcoming therapy resistance remains one of the most important unmet needs for cancer treatment. Various mechanisms contribute to the development of drug resistance in cancers, such as an increase in drug efflux, alteration of target genes, intracellular inactivation of drugs, and intracellular signaling leading to epithelial–mesenchymal transition and DNA repair. Although the role of altered metabolism in tumor cell survival and proliferation has been known for decades, the importance of metabolism in regulating the therapy response has been realized only recently. Below we highlight key studies pertaining to the role of metabolic deregulations that underline tumor resistance to therapeutic agents (Figure 2).

### 4.1. Glycolysis

One of the most well characterized mechanisms of drug resistance is related to the enhanced level and/or activity of the efflux pumps that expel the drug out of the cells. Studies show that ROS or oxidative stress regulates ATP-binding cassette (ABC) transporters, which are associated with chemoresistance [138]. The role of ABC transporters in therapy resistance has been well-reviewed [138,139]. Mechanistic analyses revealed that the activity of p-glycoprotein (Pgp, an ABC transporter), a crucial drug-efflux transporter involved in multidrug resistance (MDR), is more than doubled in prostate cancer cells exposed to acidic media (pH 6.6) [140]. The enhanced Pgp activity reduces cellular sensitivity to the cytotoxic agent daunorubicin. Similarly, the acidification of extracellular milieu reduces the cytotoxicity of weak base therapeutic agents such as doxorubicin, thus contributing drug inaction [141]. Incidentally, neutralization of tumor pH enhanced the cytotoxicity of doxorubicin, confirming the direct role of pH in regulating the tumor response to therapy. Similarly, the toxicity of paclitaxel, mitoxantrone, and topotecan is reduced in slightly acidic (pH 6.5) compared to neutral conditions (pH 7.4) [142]. In another study, the authors showed that changes in glucose levels markedly enhanced cellular ROS via NADPH oxidase 4 and thus activated HIF-1/Pgp leading to resistance to doxorubicin [143]. ROS-induced Pgp activity can be reversed by ROS scavenger NAC treatment.

Another important mechanism associated with drug resistance is enhanced DNA repair capacity of the cells. Upregulation of glycolysis is associated with enhanced repair of damaged DNA, ultimately leading to reduced sensitivity of cells to chemotherapeutic agents. A recent study showed that upregulation of glycolysis using mitochondrial respiratory modifiers protected the cancer cells from radiation-induced cytotoxicity [144]. The modifiers enhance glycolysis via induction of GLUT-1 and hexokinase, allowing uptake and utilization of glucose. The enhanced glycolysis promoted repair of radiation induced damaged DNA by activating both homologous recombination and non-homologous end joining [144]. Wagner et al. demonstrated that lactate enhances cellular DNA repair capacity by increasing the activity of DNA-PKc, leading to the protection of cells from doxorubicin (DOX) and cisplatin (CDDP) induced cytotoxicity [145]. In other studies, inhibition of glycolysis effectively overcame resistance to DNA damaging agents such as 5-fluorouracil and doxorubicin [146,147].

*Hexokinase 2*: The first enzyme of glycolysis is upregulated in various tumors, and its elevated level is associated with cisplatin resistance [148]. Inhibition of HK2 sensitizes the resistant cells to cisplatin-induced cell death and apoptosis [148,149]. Activation of ERK has been linked to the protective effect of HK2 against cisplatin. Cisplatin induces an ERK-mediated autophagic response that protects the cells from the drug-induced toxicity. In cells that overexpress HK2, enhanced autophagic response is observed, whereas, inhibition of HK2 causes suppression of autophagy, thus sensitizing resistant ovarian cancer xenografts to cisplatin [150]. In another study, Vartanian et al. demonstrated that activation of Erk by HK2 as a potential mechanism for radiation resistance in glioblastoma cells [151]. Expression of hexokinase 2 has also been implicated in tumor resistance to antimetabolites, such as 5-fluorouracil (5FU) and gemcitabine [152,153,154]. Although two inhibitors of hexokinase-2, namely 2-deoxyglucose and 3-bromopyruvate, have shown an excellent preclinical response, their clinical benefits are yet to be proved as efficient chemotherapeutic agents.

In addition to its glycolytic function, PKM2 has been shown to provide cancer cells with prosurvival and antiapoptotic properties by transcriptionally upregulating BcL-xl expression [155]. Earlier studies suggested that reduced expression of PKM2 is associated with reduced responsiveness of ovarian and colorectal cancers to platinum compounds [156,157]. However, subsequent studies demonstrated that elevated PKM2 expression is associated with reduced cisplatin sensitivity [158,159,160,161,162]. Understandably, inhibition of PKM2 in the later studies resulted in increased responsiveness of resistant cells to the drug. Similarly, contradictory observations on PKM2′s protective effect against 5FU-induced cytotoxicity has potentially impacted its use as a drug target. In colon cancer patients, high expression of PKM2 is associated with a poor response to 5FU-based therapy; however, no association was observed in gastric cancer cells [157,163]. The contradictory role in platinum-resistance suggests a cell-type dependent protective effect of PKM2 and has raised questions on the validity of PKM2 as a target for cancer therapy.

*GAPDH* is a pleiotropic enzyme whose function is dictated by its subcellular localization [164]. Localization in the nucleus regulated various non-metabolic functions of GAPDH, such as telomere protection, DNA repair, and regulation of autophagy and cell death [165]. Incidentally, two studies have reported that the presence of GAPDH enhances the sensitivity of cancer cells to therapeutic drugs. These studies demonstrated that depletion of GAPDH sensitizes tumor cells to antimetabolite agents, however, this depletion did not alter the cell’s response to other chemotherapeutic drugs, such as doxorubicin and fludarabine [166,167]. In pancreatic cancer, GAPDH translocation into the nucleus due to drug-induced oxidative stress is associated with cell death in vitro and in vivo [168]. Other studies have also demonstrated proapoptotic effect of GAPDH due to its regulation of autophagy and its interaction with one or more apoptotic cascades [165]. Although the mechanism remains unclear, the proapoptotic role of GAPDH is believed to be due to its interaction with p53. Hara et al. showed that translocation of GAPDH to the nucleus is initiated upon binding with SIAH1. The formation of complex stabilizes SIAH1 expression in the nucleus, leading to apoptosis [169,170]. p53 plays a pivotal role in stimulating the interaction between GAPDH and SIAH1 by transcriptionally upregulating the expression of both [171,172]. Evidence also suggests a direct interaction between p53 and GAPDH, wherein GAPDH enhances the proapoptotic functions of p53 through its acetylation and serine 46 phosphorylation [173]. The post-translational modification of p53 is essential for its translocation into the mitochondria to initiate Bax mediated apoptosis. The inter-regulation between GAPDH and p53 is further validated by a report highlighting that mutated p53 prevents the translocation of GAPDH into the nucleus [174]. The stabilization of GAPDH in the cytoplasm is critical for mutant p53 induced antiapoptosis. A recent study by Li et al. showed that GAPDH’s translocation into the nucleus increases transcription of p53 gene, providing additional evidence on interplay between p53 and GAPDH in regulating cellular apoptosis [175].

### 4.2. Pentose Phosphate Pathway

Many of the chemotherapeutic and targeted agents are dependent on ROS for their cytotoxicity. Due to their role in the synthesis of NADPH leading to ROS detoxification, two of the enzymes involved in oxidative PPP—glucose 6-phosphate dehydrogenase (G6PD) and 6-phosphogluconate dehydrogenase (6PGD)—have been implicated in imparting therapeutic resistance [176,177]. Analysis of doxorubicin-resistant model of colon cancer cells reveals an enhanced PPP activity and elevated levels of G6PD and glutathione [178]. The resistant cells also exhibited elevated levels of multi drug-resistant associated proteins (MRP1 and MRP2). Since the inhibition of G6PD using chemical inhibitors overcame multi drug resistance, the authors concluded that elevated glutathione levels are necessary for extruding drugs out of cells. Catanzaro et al. demonstrated that cisplatin resistant cells express elevated levels of enzyme G6PD, and the resistant cells are sensitive to G6PD inhibition [179]. Using a lung cancer model of cisplatin resistance, Hong et al. further verified that inhibition of G6PD sensitized resistant cells to cisplatin [180]. Furthermore, Zhang et al. established that the TGFβ1-FOXM1-HMGA1-TGFβ1 positive feedback loop maintain G6PD expression in cisplatin resistant cells, and that disruption of this axis sensitizes the cells to the drug [181]. Aberrant expression of 6-phosphogluconate has also been shown to be involved in chemotherapeutic and radiation resistance [182,183,184]. Although preliminary, recent evidence has shown that epidermal growth factor receptor (EGFR) phosphorylates 6PGD at tyrosine (Y481), which enhances 6PGD activity by increasing its affinity to NAD^+^. The phosphorylation also appears essential to EGFR induced radiation resistance in glioma cells [182].

The non-oxidative branch of PPP has also been implicated in chemotherapeutic resistance. Li et al. found that overexpression of Rac1 is associated with multi-drug resistance, and Rac1 mediated non-oxidative PPP is a key driver of cisplatin resistance in breast cancer cells [185]. The non-oxidative branch of PPP assists in the DNA repair process by providing the damaged cells with nucleosides. Shukla et al. identified that pancreatic cancer cells that are resistant to gemcitabine have enhanced flux of glucose carbon into the non-oxidative branch of PPP [186]. The enhanced flux is aided by increased expression of non-oxidative PPP enzymes transketolase (TKT). The HIF1α induced TKT expression in the resistant cells assist in increased pyrimidine synthesis that protects the cancer cells from gemcitabine-induced cytotoxicity [186]. Although knockdown of TKT enhances the sensitivity of cancer cells to cisplatin, in cervical cancer the underlying mechanism for that is yet to be realized [187].

## 5. Conclusions

Otto Warburg first observed the aberrant features in tumor cells characterized by a shift in their energy metabolism towards glycolysis even in conditions with ample oxygen [1]. The reprograming of tumor energetics has gained interest in the last two decades due to its association with oncogenes, tumor suppressors, and the cellular redox system [3]. A highly coordinated interplay between tumor energetics and reactive oxygen species (ROS) generates a powerful phenotype that provides the tumor cells with proliferative, antiapoptotic, and overall aggressive characteristics. Through this review, we summarized the current literature on (1) how ROS regulates tumor metabolism and (2) how metabolic adaptations in tumors regulate ROS. Albeit high levels of intracellular ROS being frequently observed in cancer cells, ROS induction is applied as a principle for most non-surgical treatments including chemotherapy and radiotherapy. By modulating the metabolic flux from oxidative phosphorylation to glycolysis and PPP, tumor cells greatly enhance its antioxidant system to maintain ROS homeostasis and prevent ROS-mediated cell death. It is no surprise that the interaction between tumor energetics and ROS plays a fundamental role in regulating tumor’s response to chemotherapeutic drugs. Though attempts of targeting tumor energetics have shown promising preclinical outcomes, the clinical benefits are yet to be fully achieved [188]. A key missing link in realizing metabolic abnormalities as a druggable target would be to understand if outcomes of altered energetics involve ROS modulation. For example, the hexokinase 2 induced autophagic response is known to involve ROS generation [189]. Since hexokinase 2 induced autophagy is associated with drug-resistance, a potential approach would include targeting metabolism with inhibitors while disabling antioxidant systems induced by radio- or chemotherapy to improve patient outcome [150]. Another important avenue to explore is to delineate the personalization of metabolic inhibitors. Since the metabolic enzymes may preferentially benefit certain cancers, a personalized therapeutic approach that involves validating the importance of the enzymes in a specific context, e.g., cancer types, may be a viable area to discover.
